# Enhancement of Neurocognitive Assessments Using Smartphone Capabilities: Systematic Review

**DOI:** 10.2196/15517

**Published:** 2020-06-24

**Authors:** John Michael Templeton, Christian Poellabauer, Sandra Schneider

**Affiliations:** 1 Department of Computer Science and Engineering University of Notre Dame Notre Dame, IN United States; 2 Department of Communicative Sciences and Disorders Saint Mary's College Notre Dame, IN United States

**Keywords:** mobile phone, mobile health, neurocognitive tests, neurodegenerative disease, neurocognitive disorders

## Abstract

**Background:**

Comprehensive exams such as the Dean-Woodcock Neuropsychological Assessment System, the Global Deterioration Scale, and the Boston Diagnostic Aphasia Examination are the gold standard for doctors and clinicians in the preliminary assessment and monitoring of neurocognitive function in conditions such as neurodegenerative diseases and acquired brain injuries (ABIs). In recent years, there has been an increased focus on implementing these exams on mobile devices to benefit from their configurable built-in sensors, in addition to scoring, interpretation, and storage capabilities. As smartphones become more accepted in health care among both users and clinicians, the ability to use device information (eg, device position, screen interactions, and app usage) for subject monitoring also increases. Sensor-based assessments (eg, functional gait using a mobile device’s accelerometer and/or gyroscope or collection of speech samples using recordings from the device’s microphone) include the potential for enhanced information for diagnoses of neurological conditions; mapping the development of these conditions over time; and monitoring efficient, evidence-based rehabilitation programs.

**Objective:**

This paper provides an overview of neurocognitive conditions and relevant functions of interest, analysis of recent results using smartphone and/or tablet built-in sensor information for the assessment of these different neurocognitive conditions, and how human-device interactions and the assessment and monitoring of these neurocognitive functions can be enhanced for both the patient and health care provider.

**Methods:**

This survey presents a review of current mobile technological capabilities to enhance the assessment of various neurocognitive conditions, including both neurodegenerative diseases and ABIs. It explores how device features can be configured for assessments as well as the enhanced capability and data monitoring that will arise due to the addition of these features. It also recognizes the challenges that will be apparent with the transfer of these current assessments to mobile devices.

**Results:**

Built-in sensor information on mobile devices is found to provide information that can enhance neurocognitive assessment and monitoring across all functional categories. Configurations of positional sensors (eg, accelerometer, gyroscope, and GPS), media sensors (eg, microphone and camera), inherent sensors (eg, device timer), and participatory user-device interactions (eg, screen interactions, metadata input, app usage, and device lock and unlock) are all helpful for assessing these functions for the purposes of training, monitoring, diagnosis, or rehabilitation.

**Conclusions:**

This survey discusses some of the many opportunities and challenges of implementing configured built-in sensors on mobile devices to enhance assessments and monitoring of neurocognitive functions as well as disease progression across neurodegenerative and acquired neurological conditions.

## Introduction

In recent history, a crossover between the fields of personal health care and mobile technology has been developed [1]. According to a 2015 US national survey on health-related apps among mobile phone owners [2], over 58% of participants had downloaded a health-related mobile app to focus on health, fitness, or medical care. This suggests that people with mobile devices not only care about their health but are also willing to use their mobile technology to help track and monitor their health in a multitude of ways. Similarly, a study [3] depicts both the American Physical Therapy Association and American Occupational Association advocating the integration of mobile health apps and systems into clinical practice, suggesting that mobile technology is also gaining clinical traction and relevance. As mobile devices become more commonplace in the health space, the formation of new and more robust health apps should be a focus.

This paper aims to provide a systematic analysis by (1) providing background on neurocognitive conditions, functional areas, and their subcategories; (2) understanding mobile technology for the purpose of updating and enhancing traditional assessment tools; (3) discussing challenges and opportunities; and (4) providing a description of a comprehensive mobile assessment tool that both individuals and clinicians can use to monitor wellness and/or decline with respect to neurocognitive function.

In this paper, we follow the Merriam-Webster’s medical definition of neurocognition: “of, relating to, or involving cognitive functioning and associated structures and processes of the central nervous system (the part of the nervous system which in vertebrates consists of the brain and spinal cord, to which sensory impulses are transmitted and from which motor impulses pass out, and which supervises and coordinates the activity of the entire nervous system).” Note that many neurological diseases and conditions yield subsequent cognitive impairments, and functional tests monitor both neurological and cognitive processes. *Neurocognitive* allows for the description of both.

**Figure 1:**
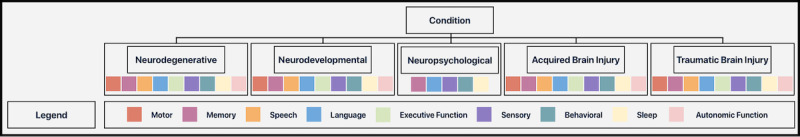
Neurological conditions and the neurocognitive functions they may affect.

Neurocognitive assessments are relevant and necessary for evaluating and monitoring neurological diseases across the categories of neurodegenerative [4], neurodevelopmental [5], neuropsychological [6], and traumatic brain injuries (TBIs) [7] or acquired brain injuries (ABIs) [8]. Neurodegenerative conditions present with progressive degeneration of neurons and neural structures. Examples include Parkinson disease, dementia, and amyotrophic lateral sclerosis [4]. Neurodevelopmental conditions (eg, autism spectrum disorders, Down syndrome, and attention deficit hyperactivity disorder) come from complications in the development of the brain [5]. TBIs, such as concussions and chronic traumatic encephalopathy, can occur in a variety of ways [7]. ABIs include stroke and meningitis [8]. Neuropsychological conditions present with behavioral and/or emotional changes, which could be the result of brain damage or a traumatic experience (eg, depression, anxiety, and post-traumatic stress disorder) [6]. Conditions could yield similar presentations to others; however, each category has unique onset conditions. Neurological diseases and conditions and their presentations that may occur are shown in Figure 1. Note that not every condition will present with all the features of that specific disorder. Combinations of symptoms may manifest depending on the individual; their age; socioeconomic background; as well as the stage, severity, and progression of the disease. Regardless of the onset conditions, understanding the taxonomies of the variety of neurocognitive conditions is vital for doctors and clinicians to formulate and administer assessments for correct diagnoses, monitoring, and rehabilitation.

## Methods

### Assessment of Neurocognitive Functions

**Figure 2:**
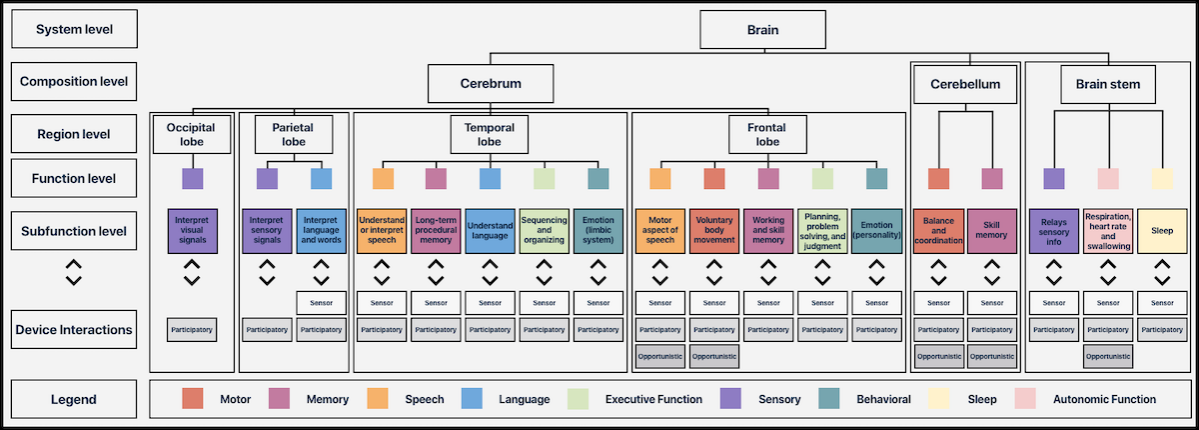
Neurocognitive breakdown into subcategories for a detailed and comprehensive assessment.

Neurocognitive functions of interest include *motor*, *memory*, *speech*, *language*, *executive function*, *sensory*, *behavioral and psychological*, *sleep*, and *autonomic functions* (Figure 1). Each of these functions correspond generally to various regions of the brain, as can be seen in Figure 2 along with their respective subfunctions. However, these brain regions are multifunctional in nature; thus, functions of interest are closely integrated with each other, the nature of which is not currently completely understood [9].

**Figure 3:**
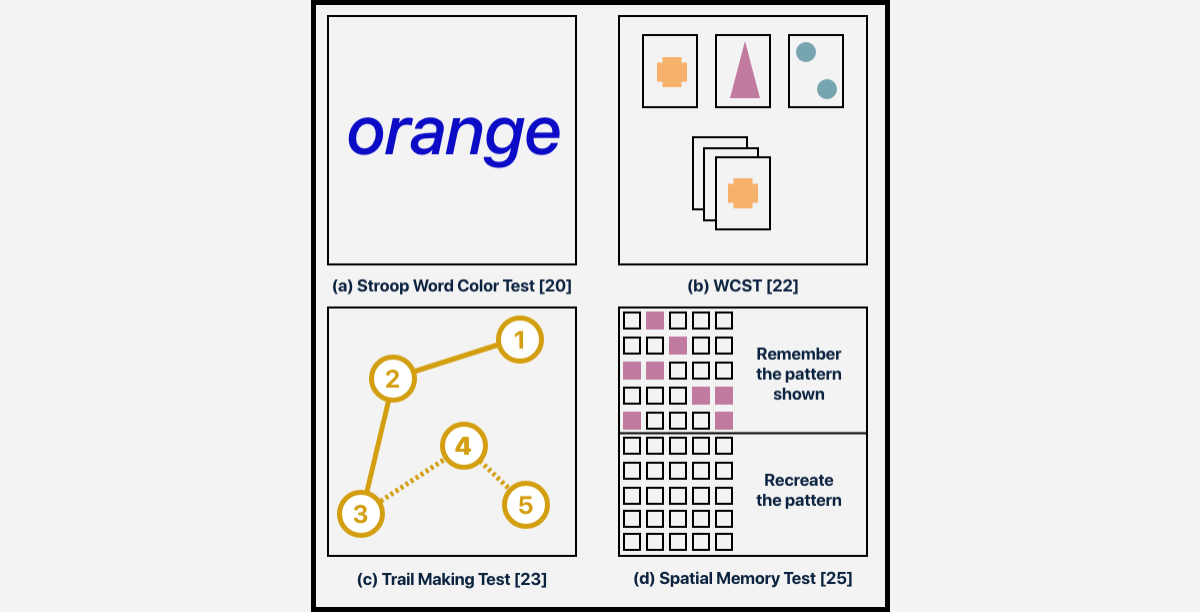
A sample view set of functional tests. WCST: Wisconsin Card Sorting Test.

There are currently formal clinical tests that can be used either for screening or assessing some of these functions of interest depicted in Table 1. Screening assessments such as the Mini-Mental Status Evaluation and Montreal Cognitive Assessment provide a quick general assessment of an individual with suspected neurocognitive impairment and identify areas needing further comprehensive evaluation. These assessments focus on a range of neurocognitive functions [10,11]. More comprehensive assessments such as the Boston Diagnostic Aphasia Examination, Dean-Woodcock Neuropsychological Assessment System, and Neurobehavioral Functioning Inventory aim to assess additional components or assess to a deeper extent [12,13]. However, none of these assessments include all functional areas of interest. A further breakdown of clinical screenings and assessments at the test level is shown in Table 2. In addition, Table 3 is a brief collection of studies and reviews across categorical neurocognitive conditions, relevant neurocognitive functions, and functional tests. Traditional testing methods for each neurocognitive function can be understood using Tables 2 and 3 and Figure 3.

As mobile devices are becoming more commonplace in neurocognitive assessments, it is necessary to review device sensors and interactions that are useful for the collection of relevant and objective data. Although some higher-end mobile devices may have additional on-device capabilities and/or sensors, currently all smartphone devices have the minimum set of capabilities listed in Table 4. Utilizing these device-based sensors and/or interactions in the formation and configuration of functional tasks enhances the usefulness and quality of the data. With the increased opportunity for user participation on their own devices and the ability of the clinician to collect and analyze enhanced objective datasets, this becomes a robust modality for the administration of these neurocognitive assessments.

**Table 1:** Current tests assessing functions of interest.

Functions	Assessments				
	MMSE^a^	MoCA^b^	BDAE^c^	NFI^d^	DWNAS^e^
Motor	X^f^	X	X	X	X
Memory	X	X	X	X	X
Speech	X	X	X	X	X
Language	X	X	X	X	X
Executive	X	X	X	X	X
Sensory	—^g^	—	—	—	X
Behavioral	—	—	—	X	X
Sleep	—	—	—	—	—
Autonomic	—	—	—	X	X

^a^MMSE: Mini-Mental Status Evaluation.

^b^MoCA: Montreal Cognitive Assessment.

^c^BDAE: Boston Diagnostic Aphasia Examination.

^d^NFI: Neurobehavioral Functioning Inventory.

^e^DWNAS: Dean-Woodcock Neuropsychological Assessment System.

^f^X denotes there is a cross-section between a clinical test and an assessment of the corresponding function.

^g^There is no cross-section between a clinical test and an assessment of the corresponding function.

**Table 2:** Test types and their functionalities.

Test	Basic functionality of test	Reference
Word Recall	Recall prompted words from memory	[14]
Reaction Time	Quantify time to recognize change in state	[15]
Static Balance	Assess stability and sway in static positions	[16]
Sit to Stand	Gross motor analysis to and from static positions	[17]
Functional Gait	Gross motor analysis of gait patterns	[18]
Apraxia Tests	Perform motor sequences across body location	[19]
Stroop Color Word Test	Assess ability to inhibit cognitive interference	[20]
Wechsler Memory Scale	Recreate visual patterns or heard sequences	[21]
Wisconsin Card Sorting Test	Sort cards based on changes in stimulus conditions	[22]
Trail Making Test	Connecting objects based on a given set of parameters	[23]
Bender-Gestalt Test	Reproduce images or patterns from various prompts	[24]
Spatial Orientation	Orientation or manipulation of objects based on direction	[25]
Boston Naming Test	Name common objects following visual cues	[26]
Syllable Repetition	Repeat various syllables or sequences	[27]

 

**Table 3:** Collection of relevant studies for traditional testing.

Publication	Category	Condition	Participants and reviews	Function(s)	Test(s)
Barbosa et al [28]	Degenerative	Parkinson’s disease	40 Parkinson’s disease and 45 control	Executive function and speech	Trail Making Test and verbal and semantic fluency
Levenson et al [29]	Degenerative	Alzheimer's disease and dementia	Review of papers	Emotion and behavioral and psychological	Structured interviews, rating scales, questionnaires, and behavioral observations
Rocchi et al [30]	Degenerative	Parkinson’s disease	27 Parkinson’s disease	Autonomic function and motor	Head‐up tilt test, Valsalva maneuver, deep breathing, and handgrip test
Czuba et al [13]	TBI^a^	TBI	108 Post TBI	Memory, motor, speech, sensory, executive function, and behavioral and psychological	Self-reporting Neurobehavioral Functioning Inventory tool (depression, somatic, memory, attention, communicate, aggression, and motor)
Whyatt et al [31]	Developmental	Autism	18 Autism, 19 control in group 1, and 22 control in group 2	Motor	Catching a ball (reflex, gross motor, and fine motor) and static balance
O’Hearn et al [32]	Developmental	Autism	Review of papers	Executive function and memory	Spatial orientation tasks, working memory, response, and inhibition
Langhorne et al [33]	ABI^b^	Stroke	Review of papers (Average of 70 subjects per trial)	Motor	Sit to Stand, standing balance, gait, gross motor (arm), and fine motor (hand)
Brady et al [34]	ABI	Stroke	Review of papers (3002 subjects)	Speech and language	Speech and language and therapies
Johnsen et al [35]	Psychological	PTSD^c^	Review of 28 studies	Speech and memory	Wechsler Memory Scale Auditory Verbal Learning Test and California Verbal Memory Test
Goldstein et al [36]	Psychological	PTSD and depression	Review of studies	Sleep and emotion	Mood Scales, diary documentation, and questionnaires

^a^TBI: traumatic brain injury.

^b^ABI: acquired brain injury.

^c^PTSD: post-traumatic stress disorder.

**Table 4:** References to previous publications regarding mobile device sensors and/or capabilities to monitor neurocognitive functions of interest.

Device Capabilities	Functions								
	Motor	Memory	Speech	Language	Executive	Sensory	Behavioral	Sleep	Autonomic
Accelerometer	Yang et al [37] and Mathie et al [38]	Yang et al [37] and Mathie et al [38]	—^a^	—	Yang et al [37] and Mathie et al [38]	—	—	Hoque et al [39] and Alqassim et al [40]	—
Gyroscope	Yang et al [37]	Yang et al [37]	—	—	Yang et al [37]	—	—	Hoque et al [39]	—
GPS	Cavallo et al [41]	—	—	—	Cavallo et al [41]	—	—	—	—
Microphone	—	Vacher et al [42]	Rosenblum et al [43] and Vacher et al [42]	Vacher et al [42]	Vacher et al [42]	—	Hossain et al [44] and Kim et al [45]	Alqassim et al [40]	Alqassim et al [40]
Camera	Zhou et al [46]	Zhou et al [46]	Rosenblum et al [43]	—	—	—	Hossain et al [44] and Nguyen et al [47]	—	Bradley et al [48]
Timer	Bhatia et al [49]	Bhatia et al [49]	Bhatia et al [49]	—	Bhatia et al [49]	—	—	Bhatia et al [49]	Bhatia et al [49]
Device interactions	Kobayashi et al [50]	Kobayashi et al [50]	—	Kobayashi et al [50]	Kobayashi et al [50]	Karuei et al [51]	—	—	—
Metadata input	—	—	—	—	—	Fries et al [52]	Fries et al [52] and Rocchi et al [53]	Fries et al [52]	Fries et al [52]
Lock, unlock and app usage	—	—	—	—	—	—	Lee et al [54] and Rocchi et al [53]	Lee et al [54] and Zhao et al [55]	—

^a^There is no cross-section between device capabilities and corresponding functions.

## Results

### Motor

#### Background and Subcategories

Completing motor tasks are often difficult for individuals with neurodegenerative conditions [56,57], neurodevelopmental conditions [31], and TBI and/or ABIs [33,58]. Motor functions can be subcategorized into fine motor, reflexes, balance, and gross motor. Traditional functional tests for the assessment of motor function are shown in Tables 3 and 5. Fine motor function testing involves the movement of the small muscle groups in one’s hands, fingers, and wrists. Methods for testing these movements include both written tests (eg, using pen and paper for trail making or writing) and object manipulation and/or interaction (eg, orienting an object in space or interacting with cards). Reflex testing requires a quick reaction motor response to an outside stimulus, which can be tactile, visual, and/or aural stimulation (eg, catching an object). Balance testing examines the user’s ability to distribute weight evenly, enabling them to remain steady. This can be examined statically (eg, standing on one leg) or dynamically (eg, going from a seated to a standing position). Gross motor function testing involves the movement of the large muscle groups for functional mobility (eg, gait). Note that although some of the tests listed in Table 5 (eg, Spatial Orientation Tests, the Trail Making Test, and the Wisconsin Card Sorting Test) are not specifically *motor* tests, the manner in which responses are collected allows the isolation of motor performance metrics.

**Table 5:** Motor functional tests and assessment methods.

Motor subcategories and functional tests			Reference
**Fine motor**			
	Apraxia Tests	[19]	
	Spatial Orientation Tests	[25]	
	Trail Making Test	[23]	
	Wisconsin Card Sorting Test	[22]	
**Reflex**			
	Apraxia Tests	[19]	
	Reaction Time Tests	[15]	
**Balance**			
	Apraxia Tests	[19]	
	Static Balance	[16]	
	Sit to Stand	[17]	
**Gross motor**			
	Apraxia Tests	[19]	
	Functional Gait Assessment	[18]	
	Spatial Orientation Tests	[25]	

#### Mobile Assessments

Mobile testing and analysis of motor functions use a variety of human-device interactions [50] and positional (eg, accelerometer, gyroscope, and GPS) [37,38,41], media (eg, camera) [46], and inherent (eg, device timer) [49] sensors, as shown in Table 4. Each motor subfunction calls for a subset of device capabilities to gain additional concrete metrics aiding in monitoring, diagnosis, and rehabilitation. Human-device interactions utilize sensors to monitor the positional state of a user’s finger during a tracing task on the screen, either via electrical current or reflection of waves. The output of this can be expressed as coordinates in 2-dimensional space and/or force measurements [50]. An example includes a geometric object (eg, circle or square) being displayed on the screen with the user’s intention to trace the shape. Relative coordinates of the trace path compared with the coordinates of the actual shape provide specific objective metrics (eg, the number of times the outline was crossed or the average distance of the trace from the outline)*.* Positional sensors (accelerometers and gyroscopes) are used to capture device motion (eg, when a user moves the device, linear and rotational motion can be assessed) [37,38]. These sensors can be helpful in enhancing object manipulation testing (eg, having the subject manipulate the mobile device itself), balance (eg, monitoring for the lack of linear and rotational motion), and gross motor function (eg, placing the device on the subject’s center of mass for gait assessment). Gross motor function can also employ the device’s GPS capabilities for additional positional information [41]. A device camera can aid in the assessment of motor function both qualitatively and quantitatively. Video analysis techniques such as slow motion or stop-action viewing can be helpful for the qualitative analysis of movement. Quantitative motion analysis of exercise activities can be performed with a detailed analysis of video recordings to analyze the subject’s movement patterns [59]. The inherent device timer allows for temporal metrics to be collected in conjunction with each of the previously mentioned metrics (eg, maximum speed, average speed, and acceleration) [49]. Finally, the sampling rate of the device’s sensors can be configured to collect additional data points as needed for an objective fine-grained analysis of the motor function.

A few mobile device apps currently being used for motor assessment include gait feedback and activity recognition. In gait rehabilitation and training, mobile device sensors were used to collect metrics for the analysis of gait patterns to establish corrective adjustments [60]. This study [60] implemented sensory feedback based on gait metrics and monitored how that feedback was interpreted for the change of subsequent steps. Similarly, mobile device sensors can aid in the classification of activities [61]. Requiring a subject to wear a smartphone on their waist to collect accelerometer and gyroscope data while various activities are performed allows for activity classification metrics to be collected.

### Memory

#### Background and Subcategories

Memory is another sector of neurocognitive assessment prominent in neurodegenerative conditions [62,63], neurodevelopmental conditions [64], TBIs or ABIs [65], and neuropsychological conditions [35]. Memory analysis can be broken down into short-term or working memory, long-term memory, and skill memory. Natural fluctuations in memory based on stress and/or fatigue are normal; however, continual trends over time showing overall decline are important for the diagnosis of diseases. Traditional functional tests regarding memory functions are presented in Tables 3 and 6. Short-term or working memory is the ability to maintain a small amount of basic information for a short period. User comprehension of a simple set of instructions, remembering visual patterns or auditory cues, are all ways in which short-term or working memory can be assessed. Long-term memory is the ability to maintain information over a long period. This information can be provided to the user via verbal, visual, or written modes. Assessment of this information could include recalling an event from a user’s past (eg, episodic memory) or could require the user to memorize information for a later assessment. Skill memory requires the individual to carry out normal functions and/or interactions without requiring much thought (eg, riding a bike or driving a car).

**Table 6:** Memory functional tests and assessment methods.

Memory subcategories and functional tests		Reference
**Short-term or working**		
	Bender-Gestalt Test	[24]
	Spatial Orientation Tests	[25]
	Wechsler Memory Scale	[21]
	Wisconsin Card Sorting Test	[22]
**Long-term**		
	Spatial Orientation Tests	[25]
	Wechsler Memory Scale	[21]
	Word Recall Test	[14]
**Skill**		
	Apraxia Tests	[19]

#### Mobile Assessments

Mobile testing and analysis of memory function make use of participatory device interactions. Human-device interactions [50], device microphones [42], and inherent [49] sensors are used for the assessment of subfunctions of short-term and long-term memory, as the user must engage with the device providing information they are intended to remember (Table 4). Human-device interactions for memory function could be used to help depict the number of times a user interacts with the screen to gather necessary information to complete a task for short- or long-term memory assessment. A mobile device enhancement of a spatial orientation game, as seen in Figure 3, comprised screen interactions notating visual cues to depict the user flipping over a card to match an original pattern. Media sensors with speech recognition capabilities can also be used for both short-term and long-term memory assessments, such as word or event recall (eg, using speech recognition for certain keywords). Subject interactions for the assessment of skill memory using Apraxia tests [19] may use positional [37,38] or media [46] sensors in a similar manner to motor function (Table 4). Having the subject wear the device while completing a physical skill task (eg, riding a bike) would yield positional metrics for balance and gross motor function to show their overall capability in the task. Skill memory in the form of explaining a procedure (eg, how to make a peanut butter and jelly sandwich) would require the device microphone or human-device interactions. The device timer is highly important for the assessment of memory function, helping to depict the length of time the user takes to express retained information.

A current mobile device app for monitoring memory function involves a memory game for rehabilitation and training, following an ABI [66]. This game hinges on a classic card matching concept in which the user must flip the cards over in pairs attempting to match all cards with their mates. Metrics are then analyzed with respect to user interactions in the app to track user memory.

### Speech

#### Background and Subcategories

Speech has become increasingly useful for the purpose of disease diagnostics. Variations in speech could be used as indicators of neurocognitive impairments across the categories of neurodegenerative [28], neurodevelopmental [67], and TBI and/or ABIs [68]. References to traditional speech testing methods are depicted in Tables 3 and 7. Speech analysis is typically broken down into frequency measures and their variations, stress, and repeatability. The fundamental frequencies and variations are acoustic characteristics of speech. Stress in speech is the degree of emphasis given a sound or syllable that can help distinguish the meanings of words or phrases. Repeatability in speech is the ability to replicate syllabic sequences for quickness and accuracy metrics.

**Table 7:** Speech functional tests and assessment methods.

Speech subcategories and functional tests		Reference
**Frequency**		
	Apraxia Tests	[19]
	Bosting Naming Test	[26]
	Stroop Color Word Test	[20]
**Stress**		
	Apraxia Tests	[19]
	Bosting Naming Test	[26]
	Stroop Color Word Test	[20]
**Repeatability**		
	Apraxia Tests	[19]
	Syllable Repetition Test	[27]

#### Mobile Assessments

References for speech analysis on a mobile device using the device’s built-in microphone [42,43] and/or camera [43] to gather sound recordings are shown in Table 4. When collected, these recordings can be used to analyze additional and deeper metrics of the speech sample. This can be expressed with measurable hertz values (eg, fundamental frequencies and their variations). The device’s speech recognition capabilities can be used for the assessment of syllable repetition tests or for evaluating what the user is saying. Each of these modes utilizes the device’s timer for the corresponding temporal metrics of the speech sample [49]. In the enhancement of a syllable repetition test (eg, repeating the sequence of Pa-Ta-Ka after a single deep breath), sensors detect metrics of accuracy (eg, number of correct sequences said by the user), frequency (eg, starting and ending frequencies), and time (eg, how long the user sustained the speech pattern).

Current mobile apps for speech function include the diagnosis, monitoring, and treatment of individuals with speech disorders [68,69]. Many of these speech apps are best suited for user difficulties in phonological representation, articulation, and phonotactics.

### Language

#### Background and Subcategories

Language is important for the assessment of phonology, morphology, semantics, syntax, and pragmatics. Phonology is the study of phonemes (eg, most basic speech sounds) of an individual language. Morphology is the study of words and other meaningful units of language. Semantics is the study of sentence meaning. Syntax is the study of sentences and phrases and the rules of grammar that they obey. Finally, pragmatics is the study of sentence meanings in context. These fundamental components of language are instrumental in assessing all neurocognitive classifications [67,70-72]. Language assessments look at how the user applies and arranges words, as well as connotation and context, into conversation, presupposition, implication, and overall systematic organization of these words [73]. A reference for the traditional assessment of language is given in Table 3.

#### Mobile Assessments

The references in Table 4 show how mobile assessments can evaluate language using a device’s built-in microphones [42] to gather sound recordings and speech recognition capabilities. These recordings can be analyzed for their linguistic style at each level of the language spectrum (eg, sentences in context or recall based on generated cues [73]). User-device interactions (eg, screen swipes or clicks) can be used for word ordering or comprehension tasks [50]. By enhancing a speech-language task (eg, picture description) on a mobile device, speech recognition can be used to assess word ordering, tense, and presupposition.

An example of this work in mobile language applications [74] uses short messaging services, smartphone apps, and gamification to enhance parental behavior that promotes language development in children. The work in this specific example is geared more toward parents who can implement interventions for their children; however, configurations for other neurocognitive conditions can also be formed.

### Executive Function

#### Background and Subcategories

Executive function refers to the abilities of judgment, planning, memory, efficiency, and time management and is relevant in the assessment of neurocognitive functioning and decline. A decline in executive function can be seen in neurodegenerative conditions [28,75], neurodevelopmental conditions [32], and TBI and/or ABIs [76]. Similar to memory analysis, executive function can fluctuate due to factors including stress and fatigue; however, constant decline trends in executive function can be used as an indicator for disease diagnosis. Traditional testing modes for the purpose of executive function are referenced in Tables 3 and 8.

**Table 8:** Executive function tests and assessment methods.

Executive function subcategories and functional tests		Reference
**Judgment**		
	Boston Naming Test	[26]
	Spatial Orientation Tests	[25]
	Stroop Color Word Test	[20]
	Wisconsin Card Sorting Test	[22]
**Planning**		
	Trail Making Test	[23]
	Wisconsin Card Sorting Test	[22]
**Time management**		
	Bosting Naming Test	[26]
	Wisconsin Card Sorting Test	[22]
**Efficiency**		
	Bender-Gestalt Test	[24]
	Spatial Orientation Tests	[25]
	Stroop Color Word Test	[20]
	Trail Making Test	[23]

#### Mobile Assessments

Analysis of executive functions implements human-device interactions [50] in conjunction with positional [37,38,41], media [42], and inherent [49] device sensors, as seen in Table 4. Similar to motor function, human-device interactions for the purpose of executive function utilize sensors to monitor the positional state of a user’s finger on the screen (eg, electrical current or reflection of waves). The output of this can be expressed as coordinates in 2-dimensional space [52], which provides opportunities for the enhancement of planning and efficiency tests (eg, the Trail Making Test). Positional sensors for the capture device motion [37,38] can be used in the enhancement of object manipulation tests (eg, having the subject manipulate the mobile device itself). GPS positional sensors can be used by having a person go from one place to another and seeing how long it takes them and the route they take [41]. Media sensors, with the purpose of speech recognition in enhancing the Boston Naming Test, can be used to collect accuracy metrics in the subject’s discernment of images [42]. Finally, the device timer is highly important in executive function analysis as it yields temporal metrics for the purpose of time management, efficiency, and judgment [49].

Current monitoring of executive function on mobile devices uses concrete tests from clinical practice, such as the Trail Making Test [77], and more abstract tasks such as prioritization and planning in scheduling [78]. Both methods are configurable for mobile apps; however, different timelines and tracking metrics are given as outcomes.

### Sensory

#### Background and Subcategories

Evaluation of visual, tactile, and aural senses are important in neurocognitive assessments, as these senses can be affected by neurocognitive conditions. Autonomic dysfunctions, including dizziness, sensation, and blurred vision, are all reasons that these sensory components should be monitored for an all-inclusive neurocognitive assessment. In addition, an individual’s perception of pain, or lack of feeling, is another sensory metric that is important to assess in neurodegenerative conditions [79] or TBI and/or ABIs [80]. A reference for the traditional assessment of sensory function is given in Table 3.

#### Mobile Assessments

Device capabilities for the monitoring of sensory function are shown in Table 4. Reaction to visual, tactile, or aural stimuli through participatory device interactions [51] or metadata input [52] regarding sensory functions are common modes for the analysis of this functional section. Mobile implementations for these stimulus responses can be implemented using vibrational patterns, screen display changes, or auditory sounds configured on the device. Screen interactions or device sensors can then be used to gauge user feedback on the signals based on configurations. Redesigned metadata surveys and questionnaires for the collection of user data allow the depiction of their sensory state. Mobile devices can allow more real-time reporting of these symptoms by allowing the user to label their pain levels throughout the day in conjunction with information on their current state (eg, during rehabilitation or right after sleep).

Current sensory function monitoring on mobile apps is both quantitative [60] and qualitative [81] in nature. A study [60] used visual, tactile, and aural feedback in conjunction with gait rehabilitation and training. This research evaluated the influence of sensory feedback on the gait pattern of the subject in real time, for the purpose of clinical rehabilitation of persons with gait abnormalities [60]. Qualitatively, self-management systems are used in practice to assist in rehabilitation by supporting goal setting and providing user state information and feedback [81].

### Behavioral and Psychological

#### Background and Subcategories

Behavioral and psychological function assessment is necessary, as neurocognitive conditions portray emotional changes after onset. This could present with the inability to express or understand different emotions [82] or expose changes to the person’s outlook [29]. This is relevant to assessing multiple capacities as *emotion* is an important feature of social interactions and quality of life and well-being. A reference in Table 3 is given for the traditional assessment of behavioral and psychological function. 

#### Mobile Assessments

References on the completion of behavioral and psychological monitoring using a device’s media (eg, camera and microphone) sensors [44,45,47] as well as metadata input [52,53], device lock and unlock, and app usage data [54] are provided in Table 4. Emotional state assessment can be completed using device media sensors through the analysis of speech and/or video samples [44,45,47]. Processing these samples, using machine learning approaches, can assist with the classification of the emotional state of its users. Similarly, the configuration of device labeling allows the user to provide a more real-time depiction of their state of being throughout the day [52,53]. The use of metadata inputs can also help with the monitoring of medication cycles and/or interpersonal relations in conjunction with mood or emotional behavior. Finally, metrics on device lock and unlock and app usage provide viable information for the emotional state. User reliance on technology and its correlation with interpersonal connections are relevant to monitor in conjunction with the emotional state of the user. Collecting these metrics directly from the user’s phone to an assessment app makes for an overall smart system.

According to Pavliscsak et al [83], mobile health apps for the collection of information regarding behavioral and psychological states are highly useful and successful, in addition to standard care measures through increased interactions. Mobile app questionnaires about user health status, psychosocial status, and progress toward treatment goals were implemented. Similarly, Juengst et al [84] explored the use of mobile apps for mood-related symptom tracking post TBI. Both studies looked at compliance, satisfaction, and usability measures for the validation of apps in practice. All metrics yielded high values, supporting the collection of this information via a smartphone.

### Sleep

#### Background and Subcategories

There are direct correlations between sleep abnormalities and neurocognitive diseases and conditions, making sleep a valuable component for neurocognitive analysis. This relationship occurs for all categories: neurodegenerative, neurodevelopmental, and neuropsychological conditions as well as TBIs or ABIs [36,85-87]. Individuals who have abnormalities in their sleep patterns ultimately show additional abnormalities among other functions [36].

#### Mobile Assessments

The sleep-monitoring capabilities that mobile devices contain are shown in Table 4. Positional sensors for movement in sleep [39,40], media sensors for sleep apnea [40], and the device timer for duration of sleep [49] are all helpful in monitoring sleep quality. Metadata input [52], lock and unlock, and app usage metrics [54,55] are also necessary for monitoring sleep quality. In sleep analysis, positional sensors (eg, accelerometer and gyroscope), measure device and subsequent user motion, or lack thereof, for assessment metrics. Microphone usage for breathing patterns is helpful for the monitoring of sleep apneas. Device timers in conjunction with both allow the temporal analysis of sleeping patterns for the evaluation of sleep. Similar to emotion, metrics on device lock and unlock and app usage provide viable information for sleep assessments, as user reliance on technology may have a negative correlation with sleep patterns [54]. Configured metadata input (eg, labeling) from the user can allow for consistent monitoring of their sleep over time, providing historical monitoring of sleep quality and quantity.

There are many current mobile apps for sleep monitoring and analysis [88]. These apps range in functionality but track total sleep time, duration of light or deep sleep, and time awake [88]. A study [89] used explicit interaction of the subject with a mobile app to monitor sleep duration. App functionalities include an alarm, labeling functionalities for sleep versus awake, and a rating system to gauge sleep quality [89]. Monitoring of users’ sleep behavior is done through the logging of metrics including: *set alarm time*, *scheduled wake up time*, *time of day in which the user goes to bed*, *number of times the alarm is snoozed*, *duration of the snooze*, and *time of day when the alarm is deactivated*. This study [89] suggests that providing more methods for users to track sleep behaviors increased the awareness of their sleep patterns and induced healthier habits.

### Autonomic Function

#### Background and Subcategories

Autonomic functions are processes that the body regulates unconsciously (eg, heart rate, respiration, swallowing, thermal regulation, digestion, and pupillary response). These functions may be affected by the onset of neurological conditions [90] but may be the result of drug therapy side effects [91]. A reference for the traditional assessment of autonomic function is given in Table 3.

#### Mobile Assessments

References on device capabilities for the monitoring of autonomic functions are depicted in Table 4. Device media sensors (eg, microphone [40] and camera [48]) are useful for monitoring functions such as breathing and pupillary response. Metadata input [49] is helpful regarding other autonomic functions (eg, digestion or urination) and may be relevant for drug intervention analysis. Although some mobile phones have heart rate sensors; Table 4 is a representation of functionalities and sensors that most mobile phones contain. Sound and image sample processing techniques (eg, machine learning) can be implemented on these devices for gaining metrics on the user’s autonomic state. Metadata input for the collection of additional autonomic functional information, which cannot be collected by device sensors, allows for a more comprehensive assessment of this area.

Current mobile apps for autonomic function monitoring include the evaluation of both breathing [92] and heart rate [93]. In a study [92], 3 training methods were created to see which provided the best outcomes. To establish which breathing training method worked best, formal metrics were collected in the following areas: skin conductance, heart rate, and respiratory signal-to-noise ratio, whereas perceived effectiveness and subjective preference were collected using questionnaires. Current work for in-home monitoring of acute and chronic cardiovascular disease uses mobile devices for both the collection of heart rate and physical activity data sent to a mobile phone via Bluetooth [93]. The mobile phone app is then used for analysis and long-term storage of information to measure progress and can be viewed by both the subject and clinician.

## Discussion

### Future of Mobile Neurocognitive Assessments

#### Devices

Current device capabilities can and should be explored for the future of neurocognitive assessments. Employing opportunistic approaches to monitoring (having device sensors on in the background without the need for formalized tests) allows for additional collection methods of objective data. An example of this approach would use the device’s GPS sensors in the background to gather information on daily commutes to see if patterns change over time. Understanding device limitations is another important aspect in this area, as data on the device cannot be collected endlessly. Participatory, opportunistic, and even hybridized approaches; further employment of current device capabilities, collection of objective data (eg, sensor metrics), and collection of additional metadata from the user, should all be addressed for the formation of an all-encompassing neurocognitive assessment. These mobile devices need to allow for additional wearable and/or internet of things (IoT) devices to interact with one another. Data fusion approaches, maintaining overall battery and data usage on devices, protecting user privacy, among others, are areas of concern that are important for device advancement and the future of these mobile neurocognitive assessments.

#### User Interactions

Moving neurocognitive assessments to mobile platforms for users allows them to explore, understand, and maintain another facet of their overall health. The ability to directly interact with their devices for training exercises, neurocognitive assessments, or rehabilitation purposes with regard to neurocognitive function allows users to have a sense of control and ownership of an important aspect of their health. Possessing these assessments on their mobile device affords users the ability to track their progress and see relevant longitudinal data. The functionality of these mobile devices is intended to not only make the user feel in control but also give the user paramount tools to assess their neurocognitive function compared with previous clinical versions. Concerns of the user to be addressed in the transition to mobile devices include preserving the privacy of their personal information and maintaining data and/or battery usage on their devices, while having positive and *simple interactions* for the assessment. These *simple interactions* require foresight in the creation of mobile testing versions.

#### Clinician Interactions

As neurocognitive testing becomes readily available on mobile devices, it is important to maintain clinical expertise. Clinical challenges arise, such as how the user interprets instructions and possible data quality and consistency issues (eg, in the cases of different neurological states between healthy populations and diagnosed neurologically impaired populations, test-retest problems, language barriers, or others). Similarly, when moving clinical assessments to mobile devices for additional sensor data, it is important to maintain relevant metadata on how the user feels and interprets their own symptoms, as there may be fewer interactions with clinical professionals who would administer questions, evaluate, and observe users. The clinician should use these devices to monitor the user, analyze the respective objective data, and ultimately assist in diagnosing conditions and formulating rehabilitation programs, if necessary. Concerns of the clinician include mobile device users diagnosing conditions on their own, a large influx of overall data, as well as maintaining the user’s personal information and the patient and clinician relationship. The benefits of these systems include the clinician’s prior review of objective and concise data, such that they can spend more time talking with the patient about specific or personal issues regarding their disease.

#### Wearables

Wearables and other functional sensing systems that work in conjunction with mobile devices can allow for even more vital data to be collected. Devices include, but are not limited to, smart watches or necklaces, fitness trackers, and even implantables. Wearables can be used in conjunction with mobile devices, or even separately, and both methods have their benefits and challenges. With the implementation of wearable devices into the system, an enhanced set of data can be obtained in addition to new information that the mobile device may not be able to detect on its own. This is directly related to more health-related sensors such as heart rate and oxygen saturation (SpO_2_). These data are important to be collected continually to monitor a user’s current state, exertion levels, etc.

Another benefit of wearables is the ability to obtain more data with additional accelerometers and gyroscopes. These additional sensors can allow for the collection of imperative data throughout the day, enhancing neurocognitive assessment systems. However, with this additional data from integrated wearables, there is a need to merge the collected data, specifically in the case of accelerometer and gyroscope use. Data fusion can be completed in a variety of ways, and each functional task might call for different fusion methodologies. For example, certain functional tasks such as motor function, including gait, balance, and sit-to-stand tasks, would be inherently beneficial for assessing with both mobile devices and wearables in tandem to get a more complete look as to how the individual moves in space. This can be seen by monitoring both devices’ positioning in space, thus providing a proximity component to the analysis. Other motor functional tests, however, such as fine motor skills and some reflex tests, may not have much of a response on the smartphone device depending on how the user interacts with the test (eg, if the device is lying on a table while being interacted with, the wearable becomes the primary source of data collection). It is imperative that data are collected on all devices when in active use; however, one device’s dataset could provide significant insights for certain tests. Subsequently, data fusion will be an area of focus when multiple devices are implemented in the same system.

### Overall Challenges and Opportunities

As traditional assessments move to mobile devices, multiple challenges arise that need to be considered and addressed. Challenges can occur within each functional area of assessment (eg, motor, memory, and speech). The monitoring of each functional area or respective subfunction requires unique configurations of a variety of device sensors. Each disease taxonomy could call for unique configurations. For example, children would require much different device interactions than older populations (eg, neurodevelopmental vs neurodegenerative conditions). Testing instructions (eg, size for visualization, lay language styles, and memory restrictions) pose challenges for device assessments. The formation of quality apps that are both detailed and understandable is important for both users and caregivers (as there are subsets of users with neurocognitive conditions that cannot complete these tasks on their own). In addition, as these devices are to be used outside of clinical settings, sample quality (eg, image or sound) poses challenges with lighting and background noise. This requires either an isolated environment to remove potential noise or filtering methods based on these files. In addition, distinguishing when to use a certain collection or assessment method over another across functional areas or combining neurocognitive functions for multimodal analysis remains a challenge. As certain device sensors are used across multiple functional areas, multimodal tasks are achievable (eg, The Stroop Color Word Test for both judgment and speech data), which reduce the administration time for functional analysis. The design of these tests, however, becomes more intensive as more metrics need to be collected. In addition, there are multiple functions that occur in ways that are, unfortunately, not easily monitorable by standalone mobile devices (eg, sleep as the user may not have a smartphone on their person or digestion as this process happens in a way that is not monitorable by a smartphone’s device sensors). Monitoring these functions requires the use of more inclusive IoT systems (eg, using smart-home technologies or other monitoring devices such as wearables). Other wearable opportunities include increasing the monitoring and real-time analysis of important features (eg, heart rate) and inclusion of new features (eg, galvanic skin response, temperature regulation, and pulse oximetry).

The collection of more objective data metrics is highly beneficial for both users and clinicians. Subjective biases are reduced with the implementation of these new impartial metrics. With the increased opportunity for user participation in their own devices and the ability of the clinician to collect and analyze enhanced objective datasets, this becomes a robust modality for the administration of these neurocognitive assessments. The use of mobile devices for assessment allows for more continual fine-grained monitoring and historical comparisons.

According to Furlong et al [69], there are few (approximately 3%) apps that are therapeutically beneficial for respective function monitoring. Similarly, there are numerous health apps in the app store that can measure some of the functions of interest; however, no apps measure all functions [3]. Highly specific apps for monitoring certain conditions are objectively helpful. Robust general apps, however, should be created for monitoring individuals before diagnosis. These *general* apps should be more than just screening tools before additional testing, but rather comprehensive apps. The formation of multiple monitoring and testing techniques should be completed for effectiveness comparisons. This would allow for highly standardized comprehensive assessment suites that can then feed into specific apps when necessary (eg, postdiagnosis or unique user conditions). Finally, although there are both notable challenges and opportunities proposed in this work, there are likely additional concerns that are not discussed, but equally important. As the relationship between mobile devices and health care deepens, the lists of challenges and opportunities will likely grow in tandem.

### Conclusions

The relationship between mobile devices and health care for the purpose of neurocognitive assessment is underway; however, due to the area being relatively young and the expansive possibilities of mobile technology, there are still numerous new avenues to be explored and/or enhanced. Upgrading mobile technology for these assessments and employing inherent device capabilities and human interactions will ultimately allow for a deeper understanding of neurological diseases. Configurations of current mobile sensors, new assessment approaches, addition of new sensors into the system, new expansive IoT systems, and exploration of data fusion and deep learning techniques for these assessments are all ways to further this adolescent connection between health care and mobile devices, not only to augment clinical interactions with users’ devices but also the overall purpose of objective and comprehensive neurocognitive assessments.

## Abbreviations

ABIs: acquired brain injuries
TBIs: traumatic brain injuries
IoT: internet of things
